# Perturbation of the two-component signal transduction system, BprRS, results in attenuated virulence and motility defects in *Burkholderia pseudomallei*

**DOI:** 10.1186/s12864-016-2668-4

**Published:** 2016-05-04

**Authors:** Natalie R. Lazar Adler, Elizabeth M. Allwood, Deanna Deveson Lucas, Paul Harrison, Stephen Watts, Alexandra Dimitropoulos, Puthayalai Treerat, Priyangi Alwis, Rodney J. Devenish, Mark Prescott, Brenda Govan, Ben Adler, Marina Harper, John D. Boyce

**Affiliations:** Department of Microbiology, Monash University, 19 Innovation Walk, Clayton, Victoria, 3800 Australia; Infection and Immunity Program, Monash Biomedicine Discovery Institute, Monash University, Victoria, Australia; Victorian Bioinformatics Platform, Monash University, Victoria, Australia; Department of Biochemistry and Molecular Biology, Monash University, Victoria, Australia; Department of Microbiology and Immunology, James Cook University, Townsville, Queensland Australia; Core Biotechnology Services, University of Leicester, Leicester, LE1 9HN UK; Department of Molecular Microbiology, Washington University School of Medicine, St. Louis, MO USA; Faculty of Dentistry, National University of Singapore, Singapore, Singapore

**Keywords:** *Burkholderia pseudomallei*, Two-component signal transduction, Transcriptomics, Flagella, Virulence

## Abstract

**Background:**

*Burkholderia pseudomallei* is the causative agent of melioidosis, a severe invasive disease of humans and animals. Initial screening of a *B. pseudomallei* signature-tagged mutagenesis library identified an attenuated mutant with a transposon insertion in a gene encoding the sensor component of an uncharacterised two-component signal transduction system (TCSTS), which we designated BprRS.

**Results:**

Single gene inactivation of either the response regulator gene (*bprR*) or the sensor histidine kinase gene (*bprS*) resulted in mutants with reduced swarming motility and reduced virulence in mice. However, a *bprRS* double mutant was not attenuated for virulence and displayed wild-type levels of motility. The transcriptomes of the *bprS, bprR* and *bprRS* mutants were compared with the transcriptome of the parent strain K96243. Inactivation of the entire BprRS TCSTS (*bprRS* double mutant) resulted in altered expression of only nine genes, including both *bprR* and *bprS*, five phage-related genes and *bpss0686*, encoding a putative 5, 10-methylene tetrahydromethanopterin reductase involved in one carbon metabolism. In contrast, the transcriptomes of each of the *bprR* and *bprS* single gene mutants revealed more than 70 differentially expressed genes common to both mutants, including regulatory genes and those required for flagella assembly and for the biosynthesis of the cytotoxic polyketide, malleilactone.

**Conclusions:**

Inactivation of the entire BprRS TCSTS did not alter virulence or motility and very few genes were differentially expressed indicating that the definitive BprRS regulon is relatively small. However, loss of a single component, either the sensor histidine kinase BprS or its cognate response regulator BprR, resulted in significant transcriptomic and phenotypic differences from the wild-type strain. We hypothesize that the dramatically altered phenotypes of these single mutants are the result of cross-regulation with one or more other TCSTSs and concomitant dysregulation of other key regulatory genes.

## Background

*Burkholderia pseudomallei* is a highly pathogenic Gram-negative organism and the causative agent of melioidosis, a potentially fatal infectious disease of humans and animals. The bacterium is endemic to tropical regions including South East Asia and Northern Australia; mortality rates resulting from melioidosis remain extremely high, with up to 42 % mortality in the Northeastern region of Thailand and 14 % mortality in Australia’s Northern Territory [1, 2]. Importantly, a 90 % mortality rate is associated with septic shock [3]. In Northern Australia, melioidosis accounts for 32 % of community-acquired bacteraemic pneumonia and 6 % of all bacteraemias [4], while in the Northeastern region of Thailand, the disease accounts for 20 % of all community-acquired septicaemias [5] and is the third most common cause of death from an infectious disease [2]. The complex clinical spectrum of melioidosis, the potentially rapid progression of disease and the fact that *B. pseudomallei* is innately resistant to a wide range of antimicrobial agents [6–8] makes treatment of this disease difficult.

For *B. pseudomallei* and most other opportunistic pathogens, the ability to sense external signals is critical for the transition from their environmental niche into the eukaryotic host, as well as for survival within specific niches within the host. Prokaryotic two-component signal transduction systems (TCSTS) constitute a critical set of regulators which act to sense environmental signals and respond by altering gene expression [9–11]. TCSTS generally consist of a membrane-bound sensor kinase (SK) and a cytosolic DNA-binding response regulator (RR) [11]. The SK protein senses extracellular stimuli and responds through the autophosphorylation of a specific histidine residue. This phosphoryl group is then transferred to an aspartate residue on the cytoplasmic RR leading to a conformational change that activates the RR, resulting in the altered expression of a specific set of genes [12]. TCSTS components are promising drug targets as these systems are not present in mammalian cells and inhibitors that target TCSTSs are likely to function in a manner distinct from existing antimicrobial agents, thereby providing an alternative treatment for multidrug resistant bacteria [13]. Moreover, many TCSTS regulate expression of virulence genes and therefore drugs that target TCSTS could reduce virulence without affecting bacterial viability and thus reduce the development of antimicrobial resistance during treatment regimens [14].

The genome of *B. pseudomallei* strain K96243 encodes more than 60 TCSTS [15] but only a few have been characterized including BPSL2024-5, VirAG*,* MrgRS and IrlRS. The IrlRS system is involved in the regulation of *B. pseudomallei* invasion of epithelial cells as well as heavy metal resistance. However, an *irlR* mutant was not attenuated for virulence in the C57BL/6 mouse, infant diabetic rat and Syrian hamster models [16, 17]. The MrgRS system responds to temperature, with increased expression of *mrgR* and *mrgS* observed during growth at 37 °C compared to 25 °C. This system may be involved in pathogenesis, but its role in virulence has not been specifically tested [18]. The VirAG system regulates the expression of the type VI secretion system cluster 1 (T6SS-1) during growth within macrophages. Both a *virG* mutant and a T6SS-1 mutant were attenuated for virulence [19]. The gene *bpsl2025*, encoding the SK of a TCSTS, was first identified in an in vivo hamster infection microarray study and a directed *bpsl2025* mutant was significantly attenuated in the hamster model (≥3-log increase in ID_50_) [20].

Here we characterise a TCSTS in *B. pseudomallei* that we have named BprRS. Inactivation of the entire BprRS system via inactivation of both genes had no effect on virulence or motility and RNA expression analysis of the double *bprRS* mutant revealed few changes in gene expression. However, inactivation of only one component (either *bprR* or *bprS*) led to an attenuated phenotype in both virulence and motility. High-throughput RNA sequencing (RNA-seq) comparing the transcriptomes of the *bprS* and *bprR* mutants with the parent strain of *B. pseudomallei* revealed a large number of expression changes in genes required for chemotaxis, flagella biosynthesis and production of malleilactone. Furthermore, many transcriptional regulators were also differentially expressed in the single gene mutant strains. We propose that the altered phenotypes displayed by the *bprR* and *bprS* single mutants are due to the orphaned sensor or the orphaned response regulator (respectively) engaging in cross-talk interactions with one or more of the other *B. pseudomallei* TCSTS.

## Results

### Identification of an attenuated *B. pseudomallei bprS* (*bpss0687*) signature-tagged transposon mutant

**Fig. 1:**
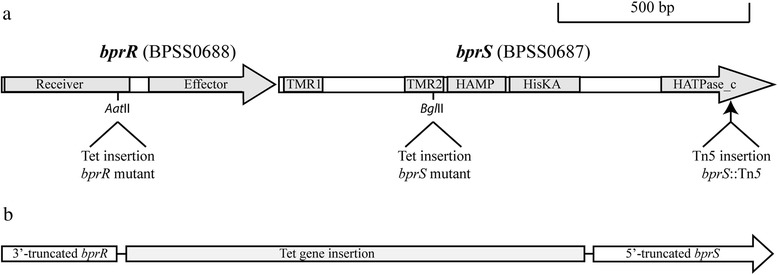
Schematic representation of the *bprR* (*bpss0688*) and *bprS* (*bpss0687*) genes in the *B. pseudomallei* K96243 wild-type strain and each of the *bprS* and *bprR* mutants. Panel **a**, organisation of the *bprRS* genes in *B. pseudomallei* wild-type showing the conserved amino acid domains encoded within each gene. Domains shown are the receiver and effector domains within *bprR* and, within *bprS*, the following domains: transmembrane regions 1 and 2 (TMR1 and 2) between which the stimulus-specific region is located, the HAMP (histidine kinases, adenylyl cyclases, methyl-accepting chemotaxis proteins, and phosphatases) signal transduction domain, the HisKA (histidine kinase A) dimerization/phosphoacceptor domain and the HATPase_c (histidine kinase-like ATPase catalytic) domain. The relative position of the insertion in each of the single mutants is shown below the gene schematic. Panel **b**, schematic representation of the regions remaining in each gene and the relative position of the tetracycline gene cassette in the *bprRS* double mutant

A signature-tagged library of 336 *B. pseudomallei* K96243 mutants was constructed and screened for reduced in vivo growth in the acute (BALB/c) mouse melioidosis model. Mutants displaying reduced hybridisation in the output pools were tested individually for an in vivo growth defect by competitive growth assays. Five mutants were identified that displayed a statistically significant in vivo growth defect (*P* < 0.001). Four of the attenuated mutants contained transposon insertions within genes required for the biosynthesis of 1,3-linked 2-*O*-acetyl-6-deoxy-β-D-manno-heptopyranose capsular polysaccharide (three in *wzm2*, one in *wcbQ*). This locus has previously been shown to be important for *B. pseudomallei* virulence [21, 22]. The fifth attenuated mutant contained a Tn*5* insertion within *bpss0687* (112 bp from the 3′ end) and this mutant was designated *bprS::*Tn*5* (Fig. 1a). The presence of a single transposon insertion in this mutant was confirmed by Southern blot analysis (data not shown). The ID_50_ of the *B. pseudomallei* parent strain K96243 and the *bprS::*Tn*5* mutant was determined in groups of five BALB/c mice following inoculation via the intranasal (i.n.) or intraperitoneal (i.p.) routes of infection. For the i.n. route of infection, the ID_50_ of the *bprS::*Tn*5* mutant was >1.3 × 10^7^ CFU while the parent strain K96243 was <1.2 × 10^4^ CFU. For the i.p. route of infection, the ID_50_ of the *bprS::*Tn*5* mutant was >1.6 × 10^4^ CFU compared with <1.2 × 10^3^ CFU for the parent strain K96243. Therefore, the *bprS::*Tn*5* mutant was attenuated for virulence via both infection routes in BALB/c mice. Importantly, the *bprS::*Tn*5* mutant displayed normal in vitro growth in both rich medium (2YT) and minimal medium (M9) indicating that the attenuated phenotype was not due to a general growth defect (data not shown).

Bioinformatic analysis of BPSS0687 revealed that the predicted protein sequence displayed significant amino acid identity to a number of characterised histidine kinases, including QseC from *Pseudomonas* spp. (92 % coverage, 39 % identity). Several conserved amino acid domains were identified within the predicted SK, including a catalytic histidine kinase-like ATPase, C-terminal domain (HATPase_c), a histidine kinase A domain (HisKA), a HAMP (histidine kinases, adenylyl cyclases, methyl-accepting chemotaxis proteins, and phosphatases) signal transduction domain and two transmembrane regions flanking the predicted stimulus-specific region (Fig. 1a). Located immediately upstream of the histidine kinase gene was a gene encoding a protein with receiver and effector domains typical of a RR (*bpss0688*). RT-PCR using primers that spanned the two genes indicated that *bpss0687* and *bpss0688* were co-transcribed (data not shown). Together these data indicate that *bpss0687* and *bpss0688* encode a cognate TCSTS pair named BprRS.

### Generation of directed *bprS*, *bprR* and *bprRS* mutants and associated virulence studies

**Fig. 2:**
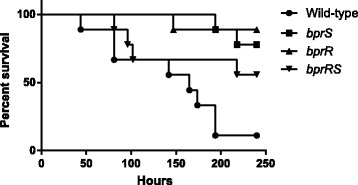
Kaplan-Meier survival curves for groups of mice infected intranasally with the *B. pseudomallei* wild-type or the *bprS, bprR or bprRS* mutant strains. Mice were infected with the following doses; wild-type strain, 6 × 10^4^ CFU; *bprS* mutant, 8 × 10^4^ CFU, *bprR* mutant, 3 × 10^4^ CFU, *bprRS* mutant 9 × 10^4^ CFU

To confirm that inactivation of *bprS* led to an attenuated phenotype, a directed *bprS* mutant was constructed using double-crossover insertional mutagenesis (Fig. 1a). We first compared the growth of the parent strain K96243 and the mutant in BALB/c mice using competitive growth assays. The *bprS* mutant displayed normal in vitro growth (data not shown) but the in vivo competitive index of the *bprS* mutant was 0.094 ± 0.08, indicative of an approximately 10-fold reduced growth rate in vivo compared to the parent strain. The attenuated phenotype was then confirmed using virulence trials in BALB/c mice (Fig. 2). Mice infected with the parent strain K96243 displayed signs of illness by 28–50 h (days 2–3) and eight of the nine mice were euthanized by 190 h (day 8) after infection. In contrast, the survival rate in the group infected with the *bprS* mutant was significantly increased (Fisher’s exact test; *P* = 0.015), with only two mice developing late stage signs of infection and required euthanasia after 190 h.

To further characterise the BprRS TCSTS, a directed *bprR* mutant (Fig. 1a) and a *bprRS* double mutant (Fig. 1b) were also constructed by double-crossover insertional mutagenesis. The *bprR* mutant was also highly attenuated for virulence (*P* = 0.003) with only a single mouse showing late-stage disease signs at 150 h (day 6) (Fig. 2). In contrast, the *bprRS* mutant was not attenuated for virulence as determined by numbers of surviving mice (Fisher’s exact test; *P* = 0.13) or time to euthanasia (Log-rank Mantel-Cox test; *P* = 0.062) (Fig. 2). These data clearly show that separate inactivation of BprRS TCSTS SK or the RR resulted in decreased virulence, but inactivation of both genes in the BprRS TCSTS system did not.

### Transcriptomic analyses of the *bprRS* double mutant

TCSTS are critical regulators of bacterial gene expression. To understand why the *bprRS* mutant was not attenuated for virulence, while the single *bprR* and *bprS* mutants were, we analysed the transcriptomes of all three mutant strains and the wild-type strain. Firstly, to identify the genes controlled by the BprRS system we compared the transcriptomes of the *bprRS* double mutant and the parent strain, K96243. In total, only nine genes were differentially expressed (five with increased and four with decreased expression) in the *bprRS* double mutant (Table 1). Both the truncated *bprR* and *bprS* gene fragments showed increased expression, suggesting that the BprRS TCSTS likely regulates its own expression. Two genes, *bpss0686* and *bpss0686a*, located immediately upstream of the *bprRS* TCSTS genes but transcribed from the other strand, showed increased expression in the *bprRS* double mutant (Table 1); these genes were also identified as differentially expressed in the *bprS* single mutant (Table 2). Bioinformatic analyses of *bpss0686a* revealed no additional information on the open reading frame and analysis of the RNA-seq data revealed that only a single sequence read of the 13 million sequence reads generated from the parent strain (K96243) matched this region, indicating that *bpss0686a* may not encode a functional protein, or it is not expressed under the conditions used for bacterial growth. Bioinformatic analysis of *bpss0686* revealed that it encoded a putative enzyme belonging to the flavin-utilizing monoxygenase super family and the nitrilotriacetate monoxgenase sub-family. Moreover, the encoded protein had 65 % identity (96 % coverage) to the 5, 10-methylene tetrahydromethanopterin reductase in *Methylobacterium extorquens* that is required for one carbon metabolism [23]. Five phage-associated genes were also differentially expressed in the *bprRS* double mutant; four of these showed decreased expression and one, encoding a phage membrane protein (*bpss1087*), showed increased expression. Three of the phage genes, *bpss1087* and *bpss1066* (encoding a predicted phage terminase/endonuclease subunits), and *bpss1072* (encoding a protein of unknown function), are located on chromosome 2 within a predicted prophage. The remaining two genes differentially expressed in the *bprRS* double mutant encoded a predicted ATPase (*bpsl0145*) and a phage membrane protein (*bpsl0146*) that were within a large region on chromosome 1 also containing a predicted prophage.

**Table 1:** Genes with differential expression in the *bprRS* double mutant compared to expression in the wild-type parent strain

Locus tag	Gene product/description	*bprRS* mutant Expression (log_2_)	FDR
Increased expression in *bprRS* double mutant relative to wild-type expression			
BPSS0686	putative 5, 10-methylenetetrahydro-methanopterin reductase	3.36	3.21E-07
BPSS0686a	protein of unknown function	5.63	7.35E-05
BPSS0687	BprS-sensor kinase protein	4.34	1.67E-07
BPSS0688	BprR-response regulator protein	2.07	1.31E-05
BPSS1087	phage membrane protein	1.13	3.41E-03
Decreased expression in *bprRS* double mutant relative to wild-type expression			
BPSL0145	protein of unknown function with ATPase domain, putative phage-encoded	−5.17	1.17E-04
BPSL0146	membrane protein	−3.48	9.28E-06
BPSS1066	phage terminase, endonuclease subunit	−3.62	2.86E-03
BPSS1072	phage-acquired protein	−4.89	9.28E-06

Genes were identified as differentially expressed if they showed >2-fold expression change (log_2_ > 1.0 or log_2_ < −1.0) compared to the wild-type strain with a False Discovery Rate (FDR) of <0.01

**Table 2:** Genes with increased expression in either the *bprR* and/or *bprS* single mutants

Locus tag	Gene name	Description	*bprS* mutant Expression (log_2_)	*bprS* mutant FDR	*bprR* mutant Expression (log_2_)	*bprR* mutant FDR
BPSL0026	*fliL*	flagellar basal body-associated protein FliL	**2.09**	**8.28E-03**	1.82#	1.52E-02
**BPSL0028**	***fliN***	**flagellar motor switch protein**	**2.25**	**5.34E-03**	**1.94**	**9.47E-03**
BPSL0031	*fliQ*	flagellar biosynthesis protein	**2.15**	**5.34E-03**	1.93#	1.08E-02
**BPSL0067**		**protein of unknown function**	**2.54**	**2.16E-05**	**2.30**	**2.20E-05**
**BPSL0068**		**putative lipoprotein**	**2.46**	**2.16E-05**	**2.30**	**2.11E-05**
BPSL0069		putative anti-sigma factor	**1.02**	**4.78E-03**	0.89	1.09E-02
**BPSL0071**		**putative catalase**	**1.31**	**4.26E-03**	**1.47**	**2.44E-03**
**BPSL0225**		**putative flagellar hook-length control protein**	**1.76**	**1.24E-03**	**1.24**	**6.62E-03**
BPSL0226	*fliJ*	flagellar fliJ protein	**1.87**	**1.75E-03**	1.34#	1.09E-02
BPSL0227	*fliI*	flagellum-specific ATP synthase	**1.94**	**5.34E-03**	1.54#	1.32E-02
BPSL0228	*fliH*	flagellar assembly protein H	**2.04**	**8.43E-03**	1.69#	1.69E-02
BPSL0230	*fliF*	flagellar MS-ring protein	**2.14**	**8.20E-03**	1.75#	1.64E-02
BPSL0231	*fliE*	flagellar hook-basal body complex protein	**1.81**	**8.84E-03**	1.39#	2.52E-02
BPSL0269	*flgA*	flagellar basal body P-ring biosynthesis protein FlgA	**2.09**	**2.38E-03**	1.58#	1.08E-02
BPSL0271	*flgC*	flagellar basal body rod protein FlgC	**2.20**	**6.66E-03**	1.75#	1.52E-02
BPSL0272	*flgD*	flagellar basal body rod modification protein	**2.12**	**7.33E-03**	1.76#	1.43E-02
BPSL0273	*flgE*	flagellar hook protein FlgE	**1.88**	**3.02E-03**	1.39#	1.22E-02
BPSL0274	*flgF*	flagellar basal body rod protein FlgF	**1.79**	**4.69E-03**	1.29#	1.66E-02
BPSL0275	*flgG*	flagellar basal body rod protein FlgG	**1.92**	**5.66E-03**	1.43#	1.64E-02
BPSL0276	*flgH*	flagellar basal body L-ring protein	**1.88**	**2.96E-03**	1.44#	1.03E-02
BPSL0278	*flgJ*	flagellar rod assembly protein/muramidase FlgJ	**1.83**	**4.51E-03**	1.27#	1.87E-02
**BPSL0403**		**mce related protein**	**1.19**	**1.26E-03**	**1.22**	**7.78E-04**
**BPSL0812**	***bpeR***	**TetR family regulatory protein**	**1.09**	**7.33E-03**	**1.08**	**8.20E-03**
**BPSL0814**	***bpeA***	**RND family acriflavine resistance protein A precursor**	**1.65**	**2.23E-03**	**1.56**	**2.22E-03**
**BPSL0815**	***bpeB***	**RND family acriflavine resistance protein**	**1.84**	**7.63E-03**	**1.79**	**7.22E-03**
BPSL1053		putative lipoprotein	0.48	3.05E-01	**1.50**	**5.74E-03**
**BPSL1112**		**putative lipoprotein**	**1.54**	**1.55E-03**	**1.24**	**6.71E-03**
BPSL1184		putative sugar-related transport, membrane protein	**1.01**	**6.70E-03**	0.88	1.37E-02
**BPSL1185**		**protein of unknown function**	**3.95**	**3.83E-05**	**4.04**	**2.11E-05**
BPSL1202		putative transport-related membrane protein	0.61	8.41E-02	**1.93**	**2.32E-04**
BPSL1203		putative carbonic anhydrase	0.63	6.17E-02	**2.03**	**9.28E-05**
**BPSL1254**		**protein of unknown function**	**1.59**	**4.26E-03**	**1.75**	**2.10E-03**
BPSL1289	*osmB*	osmotically inducible lipoprotein B precursor	0.72	3.89E-02	**1.66**	**2.80E-04**
BPSL1387		protein of unknown function	**1.09**	**1.34E-03**	0.77	8.20E-03
**BPSL1563**		**putative membrane protein of unknown function**	**1.22**	**5.17E-04**	**1.68**	**4.16E-05**
BPSL1564		putative transcriptional regulatory protein	0.50	1.17E-01	**1.37**	**9.39E-04**
BPSL1598		putative transport-related, membrane protein	0.28	3.17E-01	**1.59**	**1.66E-04**
BPSL1829		putative methyl-accepting chemotaxis protein	**1.98**	**4.26E-03**	1.34#	1.89E-02
BPSL1872		putative N-acetylmuramoyl-L-alanine amidase	1.07#	3.99E-02	**1.47**	**9.64E-03**
BPSL1931		protein of unknown function	0.83	2.35E-02	**1.01**	**9.10E-03**
BPSL2011		putative osmosis-related lipoprotein	−0.35	2.54E-01	**1.08**	**2.78E-03**
BPSL2016		protein of unknown function	0.78	5.21E-02	**1.30**	**5.35E-03**
BPSL2017		di-haem cytochrome c peroxidase	0.67	1.09E-01	**1.38**	**5.35E-03**
BPSL2193		protein of unknown function	0.90	7.52E-03	**1.12**	**2.10E-03**
BPSL2282		protein of unknown function	0.62	6.86E-02	**1.97**	**1.20E-04**
**BPSL2396**		**protein of unknown function**	**1.27**	**7.30E-03**	**1.30**	**5.74E-03**
**BPSL2397**		**protein of unknown function**	**1.46**	**2.48E-03**	**1.25**	**5.30E-03**
BPSL2398		protein of unknown function	1.12#	1.09E-02	**1.18**	**7.39E-03**
**BPSL2399**		**putative glycosyltransferase**	**1.36**	**1.55E-03**	**1.19**	**2.72E-03**
BPSL2409		ABC transporter ATP binding protein	0.85	2.82E-02	**1.09**	**8.00E-03**
BPSL2466		protein of unknown function	0.76	1.43E-02	**2.03**	**2.30E-05**
**BPSL2482**		**peptidase**	**1.25**	**2.38E-03**	**1.32**	**1.27E-03**
BPSL2483		ribosome-associated GTPase	0.97	5.86E-03	**1.01**	**4.35E-03**
**BPSL3254B**		**protein of unknown function**	**1.63**	**1.22E-03**	**1.94**	**2.75E-04**
**BPSL3291**	***fliA***	**flagellar biosynthesis sigma factor**	**1.86**	**2.45E-03**	**1.50**	**6.41E-03**
BPSL3292	*flhG*	flagellar biosynthesis protein FlhG	**2.21**	**3.79E-03**	1.74#	1.08E-02
**BPSL3293**	***flhF***	**flagellar biosynthesis regulator FlhF**	**2.23**	**1.83E-03**	**1.75**	**5.35E-03**
BPSL3294	*flhA*	flagellar biosynthesis protein FlhA	**2.09**	**7.89E-03**	1.67#	1.66E-02
BPSL3323		putative transferase	**1.55**	**8.43E-03**	1.13#	2.51E-02
BPSL3324		putative keto/oxo acyl-ACP synthase	**1.51**	**7.17E-03**	0.98	3.22E-02
BPSL3326		putative keto/oxo acyl-ACP synthase	**1.78**	**8.43E-03**	1.39#	1.99E-02
BPSL3327		putative short chain dehydrogenase	**1.74**	**3.62E-03**	1.26#	1.52E-02
BPSL3329		Rieske (2Fe-2S) domain-containing protein	**1.31**	**7.21E-03**	0.95	2.29E-02
BPSS0215	*tar*	methyl-accepting chemotaxis protein	**1.70**	**6.71E-03**	1.35#	1.58E-02
BPSS0216		putative membrane protein of unknown function	**1.12**	**4.45E-03**	0.82	1.97E-02
BPSS0223		protein of unknown function	0.82	8.44E-03	**1.44**	**2.34E-04**
BPSS0236	*ltaE*	L-allo-threonine aldolase	0.96	2.09E-02	**1.20**	**6.20E-03**
BPSS0264		protein of unknown function	1.02#	2.56E-02	**1.36**	**6.20E-03**
BPSS0298		transport related membrane protein	**1.60**	**1.36E-03**	1.07#	1.37E-02
BPSS0299	*malM*	fatty-acid CoA ligase	**2.20**	**8.23E-03**	1.63#	2.27E-02
BPSS0300	*malL*	malonyl CoA-acyl carrier protein	**2.29**	**2.38E-03**	1.60#	1.17E-02
**BPSS0301**	***malK***	**protein of unknown function**	**2.24**	**1.91E-03**	**1.60**	**9.10E-03**
BPSS0302	*malJ*	fatty acid biosynthesis-related CoA ligase	**2.30**	**2.96E-03**	1.66#	1.29E-02
**BPSS0303**	***malI***	**diaminopimelate decarboxylase**	**2.39**	**8.17E-04**	**1.81**	**2.78E-03**
**BPSS0304**	***malH***	**protein of unknown function**	**2.60**	**3.60E-04**	**1.93**	**1.38E-03**
**BPSS0305**	***malG***	**ketol-acid reductoisomerase**	**2.51**	**3.20E-04**	**1.95**	**9.39E-04**
**BPSS0306**	***malF***	**multifunctional polyketide-peptide syntase**	**2.67**	**5.32E-04**	**2.14**	**1.38E-03**
**BPSS0307**	***malE***	**gamma-aminobutyraldehyde dehydrogenase**	**2.81**	**1.53E-04**	**2.35**	**2.60E-04**
**BPSS0308**	***malD***	**protein of unknown function**	**2.75**	**1.55E-04**	**2.23**	**3.46E-04**
**BPSS0309**	***malC***	**peptide synthase regulatory protein**	**2.89**	**8.78E-05**	**2.33**	**2.10E-04**
**BPSS0310**	***malB***	**protein of unknown function**	**2.98**	**1.53E-04**	**2.62**	**2.20E-04**
**BPSS0311**	***malA***	**multifunctional polyketide-peptide syntase**	**3.10**	**5.17E-04**	**2.87**	**4.96E-04**
**BPSS0312**	***malR***	**LuxR family transcriptional regulator**	**1.47**	**6.27E-04**	**1.12**	**2.72E-03**
**BPSS0317**		**monooxygenase**	**1.54**	**1.55E-03**	**2.38**	**6.99E-05**
BPSS0325		putative membrane protein of unknown function	0.32	2.91E-01	**1.07**	**3.74E-03**
**BPSS0337**		**protein of unknown function**	**1.38**	**1.24E-03**	**1.91**	**1.20E-04**
**BPSS0623**		**outer membrane efflux protein**	**2.09**	**5.17E-04**	**2.27**	**2.10E-04**
**BPSS0624**	***macB***	**macrolide-specific ABC-type efflux carrier**	**1.95**	**1.61E-03**	**2.22**	**4.96E-04**
**BPSS0625**		**drug-efflux protein**	**1.97**	**6.27E-04**	**2.15**	**2.33E-04**
BPSS0685		protein of unknown function	**1.21**	**6.63E-04**	0.89	5.35E-03
BPSS0686^a^		Predicted 5,10-methylene tetrahydromethanopterin reductase	**3.39**	**1.14E-07**	0.35	1.71E-01
BPSS0686a^a^		protein of unknown function	**5.16**	**8.78E-05**	−0.47	5.88E-01
**BPSS0687** ^a^		**sensor kinase protein**	**5.89**	**7.90E-09**	**1.46**	**2.89E-04**
**BPSS0688** ^a^		**response regulator protein**	**4.56**	**9.34E-09**	**4.70**	**7.98E-09**
**BPSS0689**		**protein of unknown function**	**7.12**	**7.90E-09**	**7.78**	**5.57E-09**
**BPSS0690**		**protein of unknown function**	**6.45**	**1.06E-08**	**6.49**	**9.89E-09**
**BPSS0692**		**fumarylacetoacetate (FAA) hydrolase family protein**	**2.26**	**1.55E-04**	**2.16**	**2.10E-04**
**BPSS0693**		**fumarylacetoacetate (FAA) hydrolase family protein**	**2.07**	**1.53E-04**	**1.88**	**2.10E-04**
**BPSS0694**	***hpcC***	**5-carboxymethyl-2-hydroxymuconate semialdehyde dehydrogenase**	**2.14**	**2.48E-05**	**2.18**	**1.82E-05**
**BPSS0695**	***hpcB***	**3,4-dihydroxyphenylacetate 2,3-dioxygenase**	**1.81**	**8.07E-05**	**1.84**	**4.84E-05**
**BPSS0696**	***hpcD***	**5-carboxymethyl-2-hydroxymuconate delta-isomerase**	**2.19**	**1.53E-04**	**1.98**	**2.44E-04**
**BPSS0697**	***hpcG***	**2-oxo-hepta-3-ene-1,7-dioic acid hydratase**	**1.85**	**2.01E-04**	**1.89**	**1.69E-04**
**BPSS0698**	***hpcH***	**2,4-dihydroxyhept-2-ene-1,7-dioic acid aldolase**	**2.01**	**1.55E-04**	**1.99**	**1.67E-04**
BPSS0724		protein of unknown function	0.83	7.33E-03	**1.09**	**1.38E-03**
BPSS0755		LysR family regulatory protein	0.57	3.11E-02	**1.03**	**1.23E-03**
**BPSS0796A**		**H-NS-like protein**	**1.71**	**2.38E-03**	**4.39**	**1.27E-06**
BPSS0797		IclR family regulatory protein	1.03	6.40E-02	**3.95**	**1.96E-05**
BPSS0798		protein of unknown function	0.68	1.60E-01	**3.35**	**2.11E-05**
**BPSS0799**		**diguanylate phosphodiesterase**	**1.04**	**9.65E-03**	**4.48**	**1.31E-07**
BPSS0828		protein of unknown function	0.82	3.54E-02	**1.21**	**5.35E-03**
**BPSS0852**		**inosine-uridine preferring nucleoside hydrolase**	**1.60**	**6.57E-03**	**1.74**	**3.49E-03**
**BPSS0941**		**protein of unknown function**	**1.07**	**8.73E-03**	**1.33**	**2.78E-03**
**BPSS0946**	***penA***	**beta-lactamase precursor**	**1.94**	**2.38E-03**	**2.65**	**2.44E-04**
BPSS1038		protein of unknown function	0.04	9.47E-01	**1.66**	**5.01E-03**
BPSS1239		peptidase	0.33	6.92E-01	**2.76**	**2.53E-03**
BPSS1250		acetylpolyamine aminohydrolase	0.95	2.14E-02	**1.10**	**9.81E-03**
**BPSS1275**		**RNA polymerase sigma factor**	**1.25**	**7.92E-03**	**1.51**	**2.99E-03**
BPSS1296		O-methyltransferase-like protein	0.88	1.11E-02	**1.05**	**4.84E-03**
**BPSS1553**	***bprP***	**Transcriptional regulator**	**1.57**	**2.04E-03**	**1.19**	**8.84E-03**
**BPSS1554**	***bprQ***	**Protein associated with** ***bprP***	**1.43**	**1.28E-03**	**1.31**	**2.27E-03**
**BPSS1862**		**ABC transport system, ATP-binding protein**	**1.70**	**8.43E-03**	**1.71**	**7.39E-03**
BPSS1866		lipoprotein	0.21	5.80E-01	**1.44**	**2.10E-03**
BPSS1867		protein of unknown function	1.42#	1.21E-02	**2.20**	**9.10E-04**
BPSS1980		protein of unknown function	0.66	1.61E-02	**1.31**	**2.44E-04**
BPSS1996		protein of unknown function	1.13	8.44E-02	**2.84**	**8.95E-04**
BPSS2162		protein of unknown function	0.42	5.50E-01	**2.13**	**6.14E-03**
BPSS2307		amidase	0.61	1.05E-01	**1.47**	**1.92E-03**
**BPSS2308**		**protein of unknown function**	**1.01**	**1.26E-03**	**1.97**	**1.14E-05**

Genes were identified as differentially expressed if they showed >2-fold expression change (log_2_ > 1.0) compared to the wild-type strain with a False Discovery Rate (FDR) of <0.01. All significant gene expression changes are shown in bold and the locus tag, gene name and description are shown in bold when significant expression changes were observed for both mutants

^a^Increased RNA expression also observed in the *bprRS* double mutant (Table 1)

^#^Genes showing increased expression of >2-fold with a FDR of >0.01 and <0.05

### Transcriptomic analyses of the *bprS, bprR* single gene mutants

**Fig. 3:**
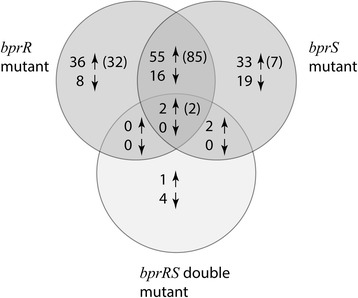
A Venn diagram showing the number of *B. pseudomallei* genes with increased expression (*up arrow*) or with reduced expression (*down arrow*) in each of the *bprR*, *bprS* and *bprRS* double mutants. Genes were identified as differentially expressed if they showed >2-fold expression change (log_2_ > 1.0) compared to the wild-type strain with a False Discovery Rate (FDR) of <0.01. The total number of genes in each mutant showing increased expression (>2-fold) with a more relaxed FDR of <0.05 are shown in brackets

In order to identify gene expression changes that resulted from perturbation of the BprRS system via inactivation of *bprS*, encoding the SK, or *bprR*, encoding the RR, we compared the transcriptomes of the *bprS* and *bprR* mutants with the wild-type strain. A total of 170 genes were differentially expressed in one or both of the single mutants and 57 genes showed increased expression and 16 reduced expression (≥2-fold change in expression at FDR <0.01) in both the *bprS* mutant and the *bprR* mutants (Fig. 3, Tables 2 and 3); thus, these gene lists show a high degree of similarity (Fisher’s exact test; *P* < 0.001). Moreover, when a slightly less stringent FDR of <0.05 was applied to the initial gene list generated, 103 of the 171 genes identified were differentially expressed in both the *bprS* mutant and the *bprR* mutant (87 increased and 16 decreased) (Fig. 3, Tables 2 and 3). Genes identified with increased expression in the *bprS* and *bprR* single mutants included many involved in the production of secondary metabolites; *bpsl3323-3329* and *bpss0689-0690* involved in fatty acid biosynthesis, *bpss0692-0698* involved in the degradation of aromatic compounds, and all the genes within the malleilactone biosynthesis locus (*bpss0298*-*bpss0312*).

**Table 3:** Genes with decreased expression in the *bprR* and/or *bprS* single mutants

Locus tag	Gene name	Gene product/description	*bprS* mutant Expression (log_2_)	*bprS* mutant FDR	*bprR* mutant Expression (log_2_)	*bprR* mutant FDR
BPSL0153		putative phage protein	**−1.37**	**8.23E-03**	−0.27	4.60E-01
BPSL0154		phage baseplate assembly protein	**−2.44**	**8.06E-03**	−0.60	3.10E-01
BPSL0155		phage baseplate assembly protein	**−1.65**	**2.96E-03**	−0.87	4.55E-02
BPSL0156		phage baseplate assembly protein	**−1.90**	**6.75E-04**	−0.77	4.26E-02
BPSL0157		phage-encoded modification methylase	**−2.80**	**8.98E-04**	−1.14	3.72E-02
BPSL0159		phage tail completion protein	−0.90	1.70E-02	**−1.72**	**1.33E-03**
BPSL0169		phage terminase, endonuclease subunit	**−2.26**	**1.02E-03**	−0.94	4.27E-02
BPSL0170		phage major capsid protein precursor	**−2.19**	**6.09E-03**	−0.94	1.08E-01
BPSL0171		putative phage capsid scaffolding protein	**−2.48**	**8.20E-03**	−0.93	1.59E-01
**BPSL0347**		**putative insertion element protein**	**−2.80**	**5.12E-03**	**−3.04**	**5.01E-03**
**BPSL0585**		**protein of unknown function**	**−1.69**	**2.38E-03**	**−2.18**	**1.04E-03**
BPSL0742		protein of unknown function	−0.67	7.95E-02	**−1.25**	**6.20E-03**
BPSL0759		protein of unknown function	**−1.25**	**9.45E-03**	−0.96	2.92E-02
BPSL0761		protein of unknown function	**−1.19**	**6.70E-03**	−0.85	2.91E-02
**BPSL1801**		**putative type-1 fimbrial protein**	**−1.23**	**4.28E-03**	**−1.89**	**2.60E-04**
BPSL2112		hydroxydechloroatrazine ethylaminohydrolase	−1.47	4.03E-02	**−2.16**	**7.22E-03**
**BPSL2113**		**putative purine catabolism-related protein**	**−1.41**	**7.63E-03**	**−2.33**	**7.13E-04**
**BPSL2114**		**protein of unknown function**	**−1.48**	**5.36E-03**	**−2.26**	**3.27E-04**
BPSL2115		ureidoglycolate hydrolase	−1.13	1.61E-02	**−1.69**	**2.22E-03**
**BPSL2116**		**allantoicase**	**−1.30**	**8.28E-03**	**−1.84**	**9.39E-04**
BPSL2117		putative uricase	−1.04	3.97E-02	**−1.73**	**3.81E-03**
**BPSL2118**		**protein of unknown function**	**−1.18**	**9.24E-03**	**−1.83**	**6.37E-04**
BPSL2508		protein of unknown function	**−1.48**	**6.03E-03**	−1.07	2.36E-02
BPSL2708		putative exported metallo-beta-lactamase-family protein	**−1.08**	**6.27E-04**	−0.59	1.62E-02
BPSL2972		IclR family regulatory protein	**−1.02**	**2.38E-03**	−0.82	8.20E-03
BPSL3171		protein of unknown function	**−1.25**	**6.57E-03**	−0.92	2.71E-02
BPSS0063	*dctD*	C4-dicarboxylate transport transcriptional response regulator	**−1.21**	**3.44E-03**	−0.95	1.32E-02
BPSS0070		IS30 transposase	−0.66	4.42E-02	**−1.06**	**6.19E-03**
BPSS0169		protein of unknown function	**−1.72**	**7.67E-03**	−1.44	1.90E-02
**BPSS0515**		**type VI secretion-associated protein, ImpA family**	**−1.86**	**2.63E-03**	**−1.75**	**5.74E-03**
**BPSS0516**		**type VI secretion protein**	**−1.79**	**8.28E-03**	**−1.98**	**9.46E-03**
**BPSS0517**	***EvpB***	**protein of unknown function**	**−2.03**	**1.85E-04**	**−1.76**	**5.84E-04**
**BPSS0518**		**type VI secretion protein**	**−1.98**	**5.17E-04**	**−1.74**	**1.41E-03**
**BPSS0520**		**type VI secretion protein**	**−1.36**	**9.44E-04**	**−1.27**	**2.01E-03**
**BPSS1034**		**protein of unknown function**	**−1.12**	**7.89E-03**	**−1.69**	**8.11E-04**
**BPSS1035**		**protein of unknown function**	**−1.74**	**5.66E-03**	**−2.24**	**2.78E-03**
BPSS1080		bacteriophage baseplate assembly protein J	**−1.47**	**8.24E-03**	−0.54	1.90E-01
BPSS1085		bacteriophage major tail tube protein	**−1.47**	**7.30E-03**	−0.75	8.02E-02
BPSS1588		protein of unknown function	−1.57	3.16E-02	**−2.21**	**5.01E-03**
BPSS1740	*lipB*	lipase chaperone	−0.83	3.87E-02	**−1.41**	**5.01E-03**
**BPSS2136**		**Family S43 non-peptidase protein**	**−1.24**	**2.38E-03**	**−1.16**	**3.97E-03**
**BPSS2138**	***oppD***	**oligopeptide transport ATP-binding ABC transport protein**	**−1.11**	**9.63E-03**	**−1.20**	**9.13E-03**
BPSS2296		transport protein	**−2.21**	**3.62E-03**	−1.87	1.14E-02

Genes were identified as differentially expressed if they showed >2-fold expression change (log_2_ < −1.0) compared to the wild-type strain with a False Discovery Rate (FDR) of <0.01. All significant gene expression changes are shown in bold and the locus tag, gene name and description are shown in bold when significant expression changes were observed for both mutants

Two genes encoding methyl-accepting chemotaxis proteins (MCPs) (*bpsl1829* and *tar*) and at least 19 genes required for flagella biosynthesis (including *fliA* encoding the flagella biosynthesis sigma factor) showed increased expression in the *bprS* single mutant; the majority of these also showed increased expression in the *bprR* mutant although mostly with FDR values between 0.01 and 0.05 (Tables 2 and 4). Genes involved in antibiotic resistance were also increased in expression and included *bpeR* (encoding a TetR family regulator), *bpeA* and *bpeB* that together encode the resistance-nodulation-division (RND) multidrug efflux pump BpeAB-OprA [24]. Other genes involved in antibiotic resistance that were over-expressed included the gene *bpsl2708*, encoding a putative metallo-β-lactamase, and *penA* that encodes a class A β-lactamase conferring resistance to ceftazidime [25, 26]. Three genes (*bpss0623*, *macB* and *bpss0625*) encoding an ABC transporter/type I secretion system predicted to be involved in drug resistance [27] were also over-expressed in the mutants. Genes with decreased expression in both the *bprR* and *bprS* mutant included five genes (*bpss0515-bpss0520*) located within the type 6 secretion system cluster 2 (T6SS-2). This cluster encodes one of six T6SSs produced by *B. pseudomallei* (designated T6SS-1through to T6SS-6) but only T6SS-1 has been determined to have a role in virulence [28].

**Table 4:** Regulatory genes differentially expressed in the *bprR* and/or *bprS* single mutants

Locus tag/gene	Description/predicted function	Expression in *bprS* mutant	Expression in *bprR* mutant		
Two component signal transduction systems				Class/Function	
BPSL1829	methyl-accepting chemotaxis protein	Increased	Increased#	MCP/Chemotaxis	
BPSS0063 *dctD*	C4-dicarboxylate transport protein	Decreased	NS	RR/Receiver	
BPSS0215 *tar*	methyl-accepting chemotaxis protein	Increased	Increased#	MCP/Chemotaxis	
BPSS0687 *bprS*	sensor kinase protein	Increased	Increased	HK/Transmitter	
BPSS0688 *bprR*	response regulator protein	Increased	Increased	RR/Receiver	
One component regulators				Domain-input	Domain-output
BPSL0812 *bpeR*	TetR family regulatory protein	Increased	Increased	Not known	TetR_N-DNA binding
BPSL1564	transcriptional regulatory protein	NS	Increased	Not known	HTH_26-DNA binding
BPSL2972	IclR family regulatory protein	Decreased	NS	IclR/Small-molecule binding	HTH_IclR-DNA binding
BPSL3291 *fliA*	flagellar biosynthesis sigma factor	Increased	Increased	Not known	Sigma70 -DNA binding
BPSS0070	IS30 transposase	NS	Decreased	Not known	HTH_38-DNA binding
BPSS0312 *malR*	LuxR transcriptional regulator	Increased	Increased	Autoind_bind- Small-molecule binding	GerE-DNA binding
BPSS0755	LysR family regulatory protein	NS	Increased	LysR_substrate- Small-molecule binding	HTH_1-DNA binding
BPSS0797	IclR family regulatory protein	NS	Increased	IclR/Small-molecule binding	HTH_IclR-DNA binding
BPSS0799	Diguanylate phosphodiesterase	Increased	Increased		EAL/Di-guanylate cyclase
BPSS1553 *bprP*	Transcriptional regulator	Increased	Increased		Trans_reg_C/DNA-binding

Genes were identified as differentially expressed if they showed >2-fold expression change (log_2_ < −1.0) compared to the wild-type strain with a False Discovery Rate (FDR) of <0.01

^#^Genes showing increased expression of >2-fold with a FDR of >0.01 and <0.05

Importantly, a large number of regulatory genes were identified as differentially expressed when the BprRS system was perturbed by single gene inactivation. Regulatory genes with increased expression in both the *bprS* and *bprR* single mutants included *fliA* and *bpeR* (discussed above) and *malR* (*bpss0312*), encoding a regulator that shares identity (97 %) with MalR in *Burkholderia thailandensis*, an orphan LuxR homolog that activates the malleilactone biosynthesis genes independently of acyl-homoserine lactone and quorum sensing systems [29, 30]. The genes *bprP* (*bpss1553*), encoding a transmembrane regulator, and the adjacent gene *bprQ*, encoding a transmembrane protein, involved in the control of the BsaN virulence regulon were also over-expressed. Importantly, the BsaN virulence regulon includes genes encoding the type III secretion system locus 3 (TTSS3) [31, 32]. The expression of *bpss0799* in both the *bprR* and *bprS* single mutants was also increased (Table 4); the encoded protein contains an EAL domain and shares significant identity with cyclic diguanylate phosphodiesterases, which are known to control motility and biofilm formation in a number of bacteria [33]. Other regulatory genes with increased expression but in only the *bprR* mutant were *bpss0797* encoding an IclR family protein, *bpss0755* encoding a LysR family protein, and *bpsl1564* that encodes a putative transcriptional regulatory protein (Table 4). Only one regulatory gene (*bpss00070*, encoding an IS30 transposase) had decreased expression in the *bprR* mutant. Regulatory genes that were reduced in expression but only in the *bprS* single mutant included *bpsl2972,* encoding an IclR family protein, and *dctD,* encoding a C4-dicarboxylate transport transcriptional response regulator (Table 4).

### Perturbation of the BprRS TCSTS affects *B. pseudomallei* motility

**Fig. 4:**
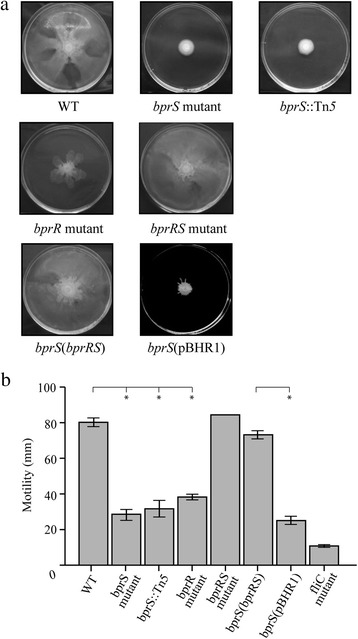
The *B. pseudomallei bprS* and *bprR* mutants display reduced motility. Swarming motility was measured on agar plates grown at 37 °C for 18 h. Panel **a**, selected images of motility plates and Panel **b**, quantification of the motility data. Strains are *B. pseudomallei* wild-type (WT), directed *bprS* mutant (*bprS*), *bprS* transposon mutant (*bprS*::Tn*5*), *bprR* mutant (*bprR*), *bprRS* double mutant (*bprRS*), complemented *bprS* mutant (*bprS*(*bprRS*)), *bprS* mutant containing empty vector (*bprS*(pBHR1)) and a non-motile *fliC* mutant control (panel **b** only). Data are mean ± SEM. * = *P* < 0.01

In the mutants with a single inactivation in either *bprS* or *bprR*, 21 genes involved in flagella biosynthesis were increased in expression, located in four different locations on chromosome 1 (Table 2). In addition, two genes encoding methyl-accepting chemotaxis proteins (*bpsl1829* and *tar*) showed increased expression, and there was decreased expression of the type-1 fimbrial gene, *bpsl1801* (Table 3) predicted to be involved in attachment to surfaces. Accordingly, we analysed the *bprS* mutants (*bprS*::Tn*5* and the *bprS* directed mutant), the *bprR* directed mutant, the parent strain K96243, as well as the *bprRS* double mutant, for their ability to migrate or swarm in a coordinated manner on solid medium. The parent strain K96243 and the *bprRS* double mutant both demonstrated a capacity for rapid swarming, with the leading edge of the swarming population migrating to the edge of the agar plate (diameter 80 mm) after 18 h at 37 °C (Fig. 4). However, all strains with either *bprS* or *bprR* inactivated demonstrated a significantly reduced ability to swarm in a coordinated manner (Fig. 4). To confirm that the reduced swarming motility was due to perturbation of the BprRS system, we complemented the *bprS* directed mutant with the complete *bprRS* operon *in trans*. Interestingly, we were unable to clone either *bprS* or *bprR* into the multicopy plasmid pBHR1 alone. Swarming motility was fully restored in the *bprS*(*bprRS*) complemented strain, whereas the *bprS* mutant harbouring empty vector (pBHR1) alone retained the reduced motility phenotype (Fig. 4). These data show that inactivation of a single component in the BprRS TCSTS can reduce the swarming ability of *B. pseudomallei*.

## Discussion

We have identified a putative TCSTS in *B. pseudomallei* strain K96243 that is encoded by *bprS* (*bpss0687*) encoding the SK, and *bprR* (*bpss0688*) encoding the RR. Inactivation of the entire BprRS system did not result in changes to virulence or motility, and RNA expression analysis of the *bprRS* double mutant identified very few genes that were differentially expressed compared to the expression in the parent strain K96243. Genes identified as differentially expressed in the *bprRS* double mutant included one (*bpss0686*) adjacent to the *bprS* gene, encoding a predicted 5, 10-methylene tetrahydromethanopterin reductase that is required for one carbon metabolism [23]. Five genes that encoded phage-related proteins were also identified. Thus, our analysis suggests that the BprRS TCSTS regulon is small but it is possible that under different growth conditions, such as during growth in a host or the environment, other genes may be identified that belong to the BprRS regulon.

In contrast, inactivation of a single component (*bprS* or *bprR*) of the BprRS TCSTS resulted in the differential expression of a large number of genes. More than 70 genes were differentially expressed in both the *bprS* and *bprR* mutant and included those involved in flagella biosynthesis, malleilactone biosynthesis, antibiotic resistance, chemotaxis and regulation. In addition, phenotypic assays showed that the *bprS* and *bprR* mutants displayed reduced growth in mice and reduced swarming motility. Together, these results clearly demonstrate that perturbation of the BprRS system, but not inactivation of the whole system, attenuates *B. pseudomallei*. We hypothesize that the changes observed as a result of perturbation of the BprRS TCSTS arise through cross-regulation with one or more of the 60 TCSTS encoded on the *B. pseudomallei* genome [15]. A number of interactions between TCSTS have been identified in *Escherichia coli*; in vitro phosphorylation assays showed that phosphorylation of RRs by non-cognate SKs was observed for at least 21 pairs [34]. While most *E. coli* cross-talk interactions are weak [34], strong cross-talk has been observed between a number of systems, including CreC and PhoB and the PmrA/B and QseB/C systems of *E. coli* [35, 36] Interactions between PhoB, PmrA/B and QseB/C happen at multiple levels including phosphorylation, dephosphorylation and DNA binding. The *E. coli* QseC SK normally acts to dephosphorylate (and deactivate) its cognate RR QseB, but QseB can be phosphorylated by PmrB at relatively high levels [36] and the binding of both RRs at the *qseBC* promoter is likely to be required for normal gene expression. In the absence of QseC the phosphorylating activity of PmrB on QseB is uncontrolled, leading to increased expression of the QseBC TCSTS and decreased expression of a range of virulence genes including those involved in pilin and flagellin synthesis. Importantly, while an *E. coli qseC* mutant was non-motile and attenuated for virulence, the single *qseB* and the double *qseBC* mutants showed wild-type virulence and motility [37]. Cross-regulation has been implicated in at least three *E. coli* TCSTS that affect flagella synthesis and motility, including predicted interactions between ArcAB and another unidentified TCSTS [38]. Therefore, we propose that in the absence of both BprR and BprS (i.e. the *bprRS* double mutant) only the expression of genes within the BprRS regulon is affected. However, in the absence of BprS, BprR is phosphorylated by one or more, non-cognate sensor kinases and interacts with genes outside the BprRS regulon. Conversely, we predict that in the absence of BprR, BprS phosphorylates one or more, non-cognate response regulators. Moreover, given that a large subset of genes was differentially expressed in both the *bprR* and *bprS* mutants, we propose that the majority of cross-talk occurs largely with a single, as yet unidentified, TCSTS cognate pair. This cross-talk then leads to a cascade of dysregulation, including altered expression of genes encoding other regulators.

Phenotypic analysis of the mutants revealed that the *bprS* and *bprR* mutants were attenuated for swarming motility. In both mutants many flagella biosynthesis genes showed increased expression compared to the wild-type strain; suggesting that altered motility resulted from incorrect levels of flagella proteins or dysregulated timing of expression. Furthermore, genes within the flagella-associated locus, including *fliC* encoding the flagella subunit, were not changed in expression compared to that in the parent strain K96243 supporting the proposition that the altered motility of the *BprS* and *BprR* mutants may have been affected by an imbalance in the expression of the flagella biosynthesis and structural components. In *E. coli* and other bacteria, flagella synthesis involves the action of numerous and complex TCSTS interactions, and we predict similar interactions between regulatory factors is also likely to be essential for the co-ordinated movement of *B. pseudomallei*. The gene *bpss0799*, encoding a protein with an EAL domain characteristic of cyclic diguanylate phosphodiesterases, was increased in expression in both the *bprS* and *bprR* mutants. Cyclic diguanylate phosphodiesterases negatively control levels of bis-(3′-5′)-cyclic dimeric guanosine monophosphate (c-di-GMP) and, together with the positive regulator diguanylate cyclase, initiate the c-di-GMP signalling system in bacteria. The global second messenger c-di-GMP then binds to specific effector molecules that target, and subsequently affect the expression of a wide range of genes involved in cellular functions including motility, biofilm formation and the production of fimbriae [33]. A previous study identified another *B. pseudomallei* gene (*cdpA* or *bpsl1263*) as encoding the cyclic diguanylate phosphodiesterase involved in flagella function and motility [39]. However, our own bioinformatic analysis (data not shown) indicates that *bpsl1263* encodes a protein with both an EAL and a GGDEF domain, the latter domain being characteristic of diguanylate cyclases. Other regulatory genes with increased expression in the *bprS* and *bprR* mutants included two genes encoding predicted MCPs, *bpsl1829* and *tar*. MCPs are the trans-membrane sensor proteins of the chemotaxis pathway that controls bacterial movement in response to available nutrients or other environmental stimuli [40]. In *E. coli*, MCPs directly transfer phosphate to the TCSTS SK CheA that then transfers the phosphate to one of two RRs, CheY or CheB. Phosphorylated CheY interacts directly with the flagella motor and causes a directional change, whereas phosphorylated CheB acts a methylesterase on the signalling MCP and modulates the amount of signal [41]. The MCP Tar sensors induce bacterial cells to move towards aspartate/maltose and away from nickel/cobalt [42]. Bioinformatic analysis did not provide any information on the type of sensor the *bpsl1829* gene encodes, but we predict that dysregulation of the expression of either MCP is likely to have an effect on motility. Although no studies have been conducted on chemotaxis mutants in any *Burkholderia* species, a study in a close relative, *Ralstonia solanacearum*, showed that a *cheA* mutant displayed significantly reduced virulence [43].

The genes *bprP* and *bprQ,* involved in the BsaN virulence regulon, both displayed increased expression when the BprRS TCSTS was perturbed. A recent transcriptomic study of a *B. pseudomallei bsaN* deletion mutant elucidated components of the BsaN virulence-associated regulatory system [32]. It is proposed that BprP directly activates the Ara-C like regulatory protein BsaN that, together with the T3SS3 chaperone BicA, act as a dual-function regulatory complex that controls the expression of the T6SS-1 (via activation of the VirAG TCSTS) and the T3SS3 effectors (*bopA*, *bopC* and *bopE*). In addition, in the *bsaN* mutant the TTSS3 genes showed increased expression, while the genes involved in malleilactone (*bpss0303-0311*) and flagella biosynthesis (*bpsl0281*, *bpsl3319*, *bpsl3320*, *bpsl3321*) showed decreased expression [32]. In our study, we observed an increase in expression of *bprP* and *bprQ*, but not the gene encoding the chaperone BicA. We also observed an increase in expression of malleilactone-associated and flagella-associated genes, but no significant change in expression of genes associated with the T3SS3 and T6SS-1. Nevertheless, it is possible that an imbalance in the expression of the proteins that initiate the transcription of *bsaN* (BprP/BicA) may have an effect on the expression of some genes within the BsaN regulon.

Perturbation of the BprRS TCSTS reduced the in vivo fitness of *B. pseudomallei* and this may, or may not have been directly associated with the loss of motility. An initial report demonstrated that a non-flagellated *fliC* mutant constructed in *B. pseudomallei* strain 1026b was not attenuated for virulence in BALB/c mice, diabetic rats or Syrian hamsters [44, 45]. In contrast, a *fliC* mutant of a more virulent strain of *B. pseudomallei* (KHW) was attenuated for virulence in BALB/c mice [46].

## Conclusions

Phenotypic and transcriptomic analyses of a newly identified TCSTS BprRS, revealed that perturbation of the system via inactivation of the SK BprS or the RR BprR led to a reduction in virulence and reduced motility, as well as a wide range of gene expression changes. Our results support the concept that *B. pseudomallei* TCSTS regulatory networks can interact and show that selective perturbation of these systems can lead to attenuation of *B. pseudomallei*. Together our results highlight the need for further systematic studies of *B. pseudomallei* TCSTS regulation and show that targeting a single TCSTS component can attenuate virulence.

## Methods

### Bacterial strains, plasmids and culture conditions

The *B. pseudomallei* strain K96243 is a human clinical isolate obtained from Thailand [15]. *B. pseudomallei* directed mutants were generated by double cross-over insertional mutagenesis using recombinant derivatives of the λ *pir*-dependent plasmid pDM4 [47]. All pDM4 derivatives were maintained in *E. coli* SM10 λ *pir* (*thi thr leu tonA lacY supE recA* RP4-2-Tc::Mu::Km *pir*), or *E. coli* S17-1 λ *pir* (*thi pro hsdR hsdM*1 *recA* RP4-2-Tc::Mu-Km::Tn*7 pir*). For some clonings, pBlueScriptSK- (Stratagene) was used as an intermediate vector prior to transfer of the insert into pDM4. The pBlueScript derivatives were grown in *E. coli* DH5α cells (F^−^*endA1 hsdR17* (r_k_^−^m_k_^−^) *glnV44* thi-1 λ^−^*recA1 gyrA96 relA1* ∆(argF- lacZYA)U196 ϕ80d*lacZ* ∆M15). The tetracycline resistance gene *tetA(C)* was used for selection of *B. pseudomallei* mutants and was isolated from either pUTminiTn*5*Tc (Biomedal S. L., Spain) or pWH1266 [48]. For complementation, the *BprRS* operon was cloned into the plasmid pBHR1, a derivative of the mobilizable broad-host-range plasmid pBBR1 [49]. All *E. coli* strains were routinely grown on lysogeny broth (LB) medium with or without selection. *B. pseudomallei* K96243 and derivatives were grown using either LB, yeast-tryptone medium (2YT), minimal medium (M9), or Ashdown’s medium (containing 64 μg/ml gentamicin). All strains were grown at 37 °C (liquid cultures with 200 rpm agitation).

### Construction and screening of *B. pseudomallei* signature-tagged mutagenesis library

A *B. pseudomallei* signature-tagged mutagenesis library was constructed using a modified miniTn*5* transposon (Biomedal S.L., Spain). Briefly, 42 unique DNA-tags were amplified from the STM-adapted Tn*916* transposons generated by Harper et al. [50] and cloned into the unique *Not*I site of pUTminiTn*5*Tc. The 42 uniquely-tagged transposons were then separately introduced into *B. pseudomallei* by conjugation and pools of 42 mutants collected. Each STM pool was then screened for reduced in vivo growth in BALB/c mice [51]. An input pool inoculum of approximately 2 × 10^8^ CFU was used to inoculate two 8–10 week old, female BALB/c mice by the i.n. route. DNA recovered from pooled input colonies and pooled output colonies (harvested from spleen), was used to generate digoxygenin-labelled (Roche) DNA probes representing tags present in each sample. Labelled probes were then used in dot blot hybridizations to determine the presence/absence of each mutant in input and output pools as described previously [50].

### Generation of *bprS*, *bprR* and *bprRS* double cross-over mutants

The *bprS*, *bprR* and *bprRS* mutants were constructed by double cross-over insertional mutagenesis using the λ *pir*-dependent vector pDM4 [47] which carries a chloramphenicol resistance gene for selection and the counter-selectable *sacB* gene. To generate the *bprS* mutagenesis construct, an internal fragment of *bprS* was disrupted by cloning of the miniTn*5 tetA(C)* gene into the *bprS Bgl*II site. Insertion of the *tetA(C)* gene at this *Bgl*II site results in the termination of BprS translation at the second transmembrane region (Fig. 1). Briefly, the primers BAP4787 (5′ TTCGTGCTGCAGGATTATCTCGAACGCCATCC 3′) containing a *Pst*I site, and BAP4788 (5′ CGAATCTCTAGAGTGGAACGGCTCGAACAC 3′) containing an *Xba*I site, were used to amplify a 981 bp PCR product from within the *B. pseudomallei* K96243 *bprS* gene. This PCR fragment, containing an internal *Bgl*II site, was digested with *Pst*I and *Xba*I and ligated into similarly digested pDM4. Primers BAP3325 (5′ CGGCTTAGATCTAGGTCGAGGTGGCC 3′) and BAP4629 (5′ TCCACCAGATCTATTTGCCGACTACCTTGGTG 3′), both containing a *Bgl*II site, were used to amplify the miniTn*5 tetA(C)* gene. This PCR product and pDM4 containing the *bprS* internal fragment were digested with *Bgl*II and ligated to generate the plasmid pAL603 which was then introduced by transformation into *E. coli* SM10 λ *pir*.

For generation of the *bprR* mutagenesis construct, pBluescriptSK- was used as an intermediate plasmid prior to cloning into pDM4. Primers BAP6698 (5′ GAACACCCCGGGCGCGAGATCGACATGCCCGGACACGCC 3′) containing an *Xma*I site, and BAP6699 (5′ CGTCGTACTAGTACATGAAGCCGAAGCCCTCGTTGCCG 3′) containing an *Spe*I site, were used to amplify a 1648 bp PCR product from *B. pseudomallei* K96243 encompassing the entire *bprR* gene, 687 bp of upstream and 241 bp of downstream DNA. This PCR product, containing an internal *Aat*II site within *bprR* (Fig. 1a) was digested with *Xma*I and *Spe*I and ligated into similarly digested pBluescriptSK-. Primers BAP6700 (5′ AAAACAGACGTCTTGCTAACGCAGT 3′) and BAP6701 (5′ AAAAATGACGTCAGTGGTGAATCC 3′), both containing an *Aat*II site, were used to amplify the *tetA(C)* gene from pWH1266. This product and the pBluescriptSK- derivative containing the *bprR* fragment were digested with *Aat*II and ligated to generate the plasmid pBluescriptSK::*bprR*-*tet* which was then introduced into *E. coli* DH5α by transformation. This insertion of the tetracycline resistance gene at the internal *Aat*II site causes a truncation of *bprR* within the initial receiver domain (Fig. 1a). The fragment containing the disrupted *bprR* was released from pBluescriptSK::*bprR*-*tet* by digestion with *Xma*I and *Spe*I and ligated into pDM4, generating the plasmid pAL1067 which was then introduced into *E. coli* SM10 λ *pir* cells.

To generate the *bprRS* double mutant, up- and down-stream regions encompassing the 5′ and 3′ ends of *bprR* and *bprS* respectively were cloned into pBluescriptSK-, with the *tetA(C)* gene from miniTn*5* gene inserted between the two fragments. Insertion of this construct into the genome leads to deletion of a 3′ segment of *bprR* (encoding part of the receiver domain and all of the effector domain) as well as deletion of a 5′ segment of *bprS* (encoding both transmembrane regions and the HAMP and HisKA domains) (Fig. 1b). Briefly, primers BAP6704 (5′ TCGACGCCCGGGTCACCGAGCTGCTGACGATCGGC 3′) containing an *Xma*I site, and BAP6705 (5′ CGGCGCACTAGTTATCAGACCGACTACGCGCC 3′), containing a *Spe*I site, were used to amplify a 973 bp PCR fragment including the 3′ end of *bprS* and down-stream flanking DNA. This product was digested with appropriate enzymes and ligated into pBluescriptSK- digested with *Xma*I and *Spe*I and introduced into DH5α cells. Primers BAP6685 (5′ AAAACAGAATTCTTGCTAACGCAGT 3′) and BAP6686 (5′ AAAAATGAATTCAGTGGTGAATCC 3′), both containing an *Eco*RI site, were used to amplify the *tetA(C)* gene from pWH1266. This product and pBluescriptSK- containing the DNA fragment containing *bprS* and flanking region were digested with *Eco*RI, ligated and introduced into *E. coli* DH5α. Primers BAP6702 (5′ AGCGGCGGGCCCCCGCATGCACCGAGCCC 3′) containing an *Apa*I site, and BAP6703 (5′ GAGGCCATCGATTTGATCGTAGACGTCCG 3′) containing a *Cla*I site, were used to amplify a 1021 bp PCR product encompassing the 5′ end of *bprR* and up-stream flanking DNA. This product was digested with *Apa*I and *Cla*I and ligated into the similarly digested pBluescriptSK- containing *bprS* plus flanking region and the *tetA(C)* gene, and then introduced into *E. coli* DH5α cells, generating the plasmid pAL1062. This plasmid was digested with *Apa*I and *Spe*I and the fragment containing *bprR*-*tet*-*bprS* was ligated into pDM4. The resulting plasmid, designated pAL1066, was then introduced into *E. coli* SM10 λ *pir* cells.

Each of the recombinant plasmids pAL603, pAL1067 and pAL1066 was mobilised from *E. coli* SM10 λ *pir* into *B. pseudomallei* K96243 by conjugation and putative double cross-over mutants were selected on LB agar containing gentamicin (8 μg/mL) and tetracycline (25 μg/mL). All mutations were confirmed by PCR and sequence analysis (data not shown).

### Complementation of the *bprS* mutant

The *bprS* mutant was complemented by cloning an intact copy of the *bprRS* operon into the multicopy plasmid pBHR1. Primers BAP7136 (5′ CGGCGCTTTAAAGGCTCGATACTGACTGCTGCCGGC 3′) containing a *Dra*I site, and BAP7137 (5′ CGCGCGCCATGGATCGTCTGACGGCCGAAACC 3′) containing an *Nco*I site, were used to amplify a 2483 bp PCR product from *B. pseudomallei* K96243. This fragment encoded *bprS* and *bprR* as well as 353 bp of the upstream region predicted to contain the *bprRS* promoter. The PCR product was digested with *Dra*I and *Nco*I and ligated into similarly digested pBHR1, disrupting the *cat* gene and generating the plasmid pBHR1::*bprRS.* This plasmid was then introduced into *E. coli* S-17 λ *pir* and kanamycin resistant, chloramphenicol sensitive clones selected. The pBHR1::*bprRS* plasmid and empty pBHR1 (negative control) were separately mobilised from *E. coli* S-17 into the *bprS* mutant by conjugation. Recombinants containing the pBHR1::*bprRS* plasmid were selected on LB agar containing kanamycin (1000 μg/mL) and tetracycline (25 μg/mL) while those containing the empty pBHR1 plasmid were selected on LB agar containing kanamycin (1000 μg/mL), tetracycline (25 μg/mL) and chloramphenicol (40 μg/mL). Strains containing the correct plasmids were identified by PCR and nucleotide sequence analysis (data not shown). Neither *bprR* nor *bprS* alone could be cloned into the pBHR1 plasmid despite numerous attempts.

### Competitive in vivo growth assays

Overnight cultures of *B. pseudomallei* wild-type parent strain K96243 and the *bprS* mutant were grown in LB to an optical density at 600 nm (OD_600_) of 0.2. Equal volumes of K96243 and *bprS* mutant culture were combined and 20 μl (containing approximately 4 × 10^5^ CFU) of an appropriate dilution was used to infect three 6–8 week-old female BALB/c mice by the i.n. route. After 24 h, mice were euthanized in accordance with animal ethics requirements. Spleens were harvested, homogenised in phosphate-buffered saline pH = 7.2 (PBS) and plated onto LB agar. After growth at 37 °C for 24 h, 100 colonies from these in vivo growth plates and 100 colonies representing the input culture were patched onto LB agar with or without tetracycline (25 μg/ml) to determine the proportion of *bprS* mutant to K96243 wild-type bacteria. The competitive index was calculated as the proportion of mutant to wild-type bacteria recovered from the mouse spleens divided by the proportion of mutant to wild-type bacteria present in the input inoculum.

### Virulence assays

Overnight cultures of the parent strain K96243 and the *bprS*, *bprR* and *bprRS* mutants were subcultured in fresh medium with appropriate antibiotics and grown to mid-log phase to an OD_600_ of 0.8 (equivalent to ~5.0 × 10^8^ CFU/ml). For the ID_50_ assays, groups of five 8–10 week-old BALB/c mice were either inoculated i.n (1.0 × 10^6^ CFU in 20 μl volume) or injected i.p (1.0 × 10^3^ CFU in 200 μl volume) with LB containing mid-exponential phase bacteria. For the direct virulence assays, groups of nine 6–8 week-old, female BALB/c mice were infected i.n. with 20 μl doses containing approximately 5.0 × 10^4^ CFU. Mice were monitored for 10 days for signs of disease and euthanized at the end of the experiment or when moribund, in accordance with animal ethics requirements. The ID_50_ for the parent strain K96243 and mutant strain was determined using the calculation described by Reed and Muench [52] based on cumulative moribund infections. For direct virulence assays, differences in survival were calculated using Fisher’s exact test and differences in time to death determined using the log-rank Mantel-Cox test. Spleens from three mice in each group were harvested, homogenised in PBS and plated onto LB agar. The stability of the mutants was assessed by patching the recovered colonies onto agar plates with or without tetracycline (25 μg/ml). All of the mutations were stable over the time course of the experiment. The virulence of the complementation strain *bprS*(*bprRS*) could not be assessed as the complementation plasmid was highly unstable in the absence of antibiotic selection (>90 % plasmid loss over 16 h).

### RNA purification

*B. pseudomallei* parent strain K96243 and the directed *bprS, bprR* and *bprRS* mutants were grown overnight at 37 °C in LB with appropriate antibiotics (K96243 in gentamicin 64 μg/ml; mutants in gentamicin 64 μg/ml and tetracycline 25 μg/ml). Strains were subcultured 1/50 into fresh antibiotic-free medium and grown at 37 °C with shaking (200 rpm) to an OD_600_ of 0.5. The cells were harvested by centrifugation, and RNA purified using Trizol reagent (Gibco/BRL) as specified by the manufacturer. RNA was DNase treated using the TURBO DNA-free kit (Ambion), followed by processing with a QIAGEN RNeasy kit clean-up with on-column DNase digestion as per the manufacturer’s instructions.

### High-throughput RNA sequencing

Double-stranded cDNA synthesis and high-throughput RNA-seq were performed as described previously [53]. For each strain three biological replicates were sequenced. Trimmed sequence reads were aligned to the *B. pseudomallei* K96423 genome sequence using SHRiMP [54] and normalised read counts were compared using voom and limma as described previously [53]. For each replicate sample, between 6.4 million and 14.0 million sequence reads were mapped uniquely to the K96423 genome sequence. The RNA-seq data is available at the NCBI Gene Expression Omnibus; accession number GSE77970. Differentially expressed genes were identified as those with a greater than 2-fold change in expression (1.0 log_2_) across all replicates at a false discovery rate (FDR) of <0.01.

### Swarming motility assays

Swarming agar plates were prepared fresh on the day of the assay using the modified protocol of Tunpiboonsak et al. [55]. Briefly, plates were prepared using 0.5 % *w/v* agar-agar (Merck), 8 g/L nutrient broth No.2 (Oxoid) and 5 g/L D(+)- glucose (dextrose) (May and Baker) and dried carefully to give constant moisture content. Overnight cultures of *B. pseudomallei* parent strain K96243 and mutant strains were subcultured 1/50 into fresh medium with appropriate antibiotics (no selection for parent strain, 25 μg/ml tetracycline for all mutants and complemented mutants) and grown at 37 °C with shaking (200 rpm) to late exponential phase (OD_600_ of ~2.5). A 5 μl volume of each culture was spotted onto the centre of a swarming agar plate which was then incubated at 37 °C for 18 h in the dark. The diameter of the swarming population was then measured. Statistical analysis of swarming distance was determined by Student’s t-test with a *P*-value of <0.05 considered significant.

### Ethics approval and consent to participate

All animal experiments were carried out in accordance with the provisions of the “Prevention of Cruelty to Animal Act, 1986”, the “Australian Code of Practice for the Care and Use of Animals for Scientific Purposes 7th edition, 2004” and the Monash University Animal Welfare Committee Guidelines and Policies. The protocol was approved by the Monash Animal Research Platform (MARP)-2 Animal Ethics Committee (AEC) of Monash University (AEC number MARP/2011/067).

### Consent for publication

Not applicable.

### Availability of data and materials

The RNA-seq data is available at the NCBI Gene Expression Omnibus; accession number GSE77970.

## Abbreviations

2YT: yeast-tryptone medium
AEC: Animal Ethics Committee
FDR: false discovery rate
HAMP: Histidine kinases, Adenyl cyclases, Methyl-accepting proteins and Phosphatases
HATPase_c: Histidine kinase-like ATPase, C-terminal domain
HisKA: histidine kinase A domain
i.n.: intranasal
i.p.: intraperitoneal
LB: lysogeny broth
MARP: Monash Animal Research Platform
MCPs: methyl-accepting chemotaxis proteins
OD_600_: optical density at 600 nm
PBS: phosphate-buffered saline pH = 7.2
RNA-seq: RNA sequencing
RND: resistance-nodulation-division
RR: response regulator
SK: sensor kinase
T6SS-1: type VI secretion system cluster 1
T6SS-2: type 6 secretion system cluster 2
TCSTS: two-component signal transduction system
TTSS3: type III secretion system locus 3
